# *Lysmata arvoredensis* nov. sp. a new species of shrimp from the south coast of Brazil with a key to species of *Lysmata* (Caridea: Lysmatidae) recorded in the southwestern Atlantic

**DOI:** 10.7717/peerj.5561

**Published:** 2018-09-05

**Authors:** Bruno W. Giraldes, Thais P. Macedo, Manoela C. Brandão, J. Antonio Baeza, Andrea S. Freire

**Affiliations:** 1 Environmental Science Centre, Qatar University, Doha, Qatar; 2 Laboratório de Crustáceos e Plâncton, Departamento de Ecologia e Zoologia, Universidade Federal de Santa Catarina, Florianópolis, Santa Catarina, Brazil; 3 Observatoire Océanologique de Villefranche-sur-Mer, Villefranche-sur-Mer, France; 4 Department of Biological Sciences, Clemson University, Clemson, SC, USA; 5 Smithsonian Marine Station at Fort Pierce, Fort Pierce, FL, USA; 6 Departamento de Biología Marina, Facultad de Ciencias del Mar, Universidad Católica del Norte, Coquimbo, Chile

**Keywords:** Marine biodiversity, Peppermint shrimp, Maare project, REBIO do Arvoredo, Phylogeny and taxonomy, Santa Catarina, Decapod

## Abstract

*Lysmata arvoredensis* sp. nov. inhabits temperate waters in the south coast of Brazil and is named in tribute to the Marine Protected Area REBIO Arvoredo. This is the fourth species belonging to the genus *Lysmata* recorded for the region and the ninth for Brazil. *L. arvoredensis* sp. nov. can be distinguished from other species of *Lysmata* by the presence of a nearly completely fused accessory branch with a single free unguiform segment on the outer antennular flagellum; a rostrum with seven dorsal (2+5) and three ventral teeth; a stylocerite with a pointed tip bearing mesial setae; a second pereiopod with 22–24 carpal subsegments and 14–16 subsegments in the merus; a merus of the third pereiopod with five ventrolateral and 12 ventral spines on the propodus; and its color pattern, with red bands and patches in pleonites 2–3 that resemble a mask in dorsal view. Molecular characters demonstrate that *L. arvoredensis* sp. nov. is most closely related to other species of *Lysmata* belonging to the Neotropical and Cleaner clades. To support future ecological studies in the region, identification keys to the species of *Lysmata* recorded in the southwestern Atlantic Ocean are provided.

## Introduction

Shrimps belonging to the genus *Lysmata* Risso, 1816 are commonly traded in the aquarium industry (Calado et al., 2003; Baeza & Behringer, 2017) because of their beautiful coloration, ability to remove ectoparasites from reef fishes (Karplus, 2014), and capability to control pests in aquaria (Rhyne, Lin & Deal, 2004). In the last decade, the genus has received considerable attention: several new species have been described (Rhyne, Calado & Dos Santos, 2012; Gan & Li, 2016) and complexes of cryptic species have been partially resolved (Rhyne & Lin, 2006; Baeza & Behringer, 2017). Furthermore, a series of studies focusing on the phylogeny of the genus coupled with behavioral experiments have improved our understanding regarding the evolution of hermaphroditism in caridean shrimps (Baeza, 2009, 2010, 2013; Fiedler et al., 2010; Baeza & Fuentes, 2013; De Grave et al., 2014). Currently, a total of 45 species are recognized worldwide (De Grave & Fransen, 2011; Rhyne, Calado & Dos Santos, 2012; Soledade et al., 2013; Gan & Li, 2016; Prakash & Baeza, 2017; De Grave & Anker, 2018) and eight of them have been recorded in Brazil: *L. ankeri* Rhyne & Lin, 2006 and *L. bahia* Rhyne & Lin, 2006 previously misidentified as *L. wurdemanni* (Gibbes, 1850) (Rhyne & Lin, 2006); *L. grabhami* (Gordon, 1935) previously misidentified as *L. amboinensis* (de Man, 1888) (Kassuga, Diele & Hostim-Silva, 2015); *L. moorei* (Rathbun, 1901) (Coelho et al., 2006; Coelho Filho, 2006); *L. jundalini* Rhyne, Calado & Dos Santos, 2012 previously misidentified as *L.* cf. *intermedia* (Kingsley, 1878) (Rhyne, Calado & Dos Santos, 2012; Terossi et al., 2018); the Indo-Pacific *L. vittata* (Stimpson, 1860) improperly described as a new species (i.e., *L. rauli* Laubenheimer & Rhyne, 2010) (Laubenheimer & Rhyne, 2010; Soledade et al., 2013); *Lysmata* cf. *lipkei* Okuno & Fiedler, 2010, described from Japan (Okuno & Fiedler, 2010) and likely representing a second nonindigenous species in the region (Pachelle et al., 2016); and *L. wurdemanni* (Gibbes, 1850) (Terossi et al., 2018).

In this study, we describe a new species of *Lysmata* from the south coast of Brazil. To support future ecological studies in the southwestern Atlantic Ocean, identification keys to species belonging to the genus *Lysmata* present in Brazil are provided. One key is based on morphology and a second key is based on color pattern.

## Material and Methods

**Figure 1:**
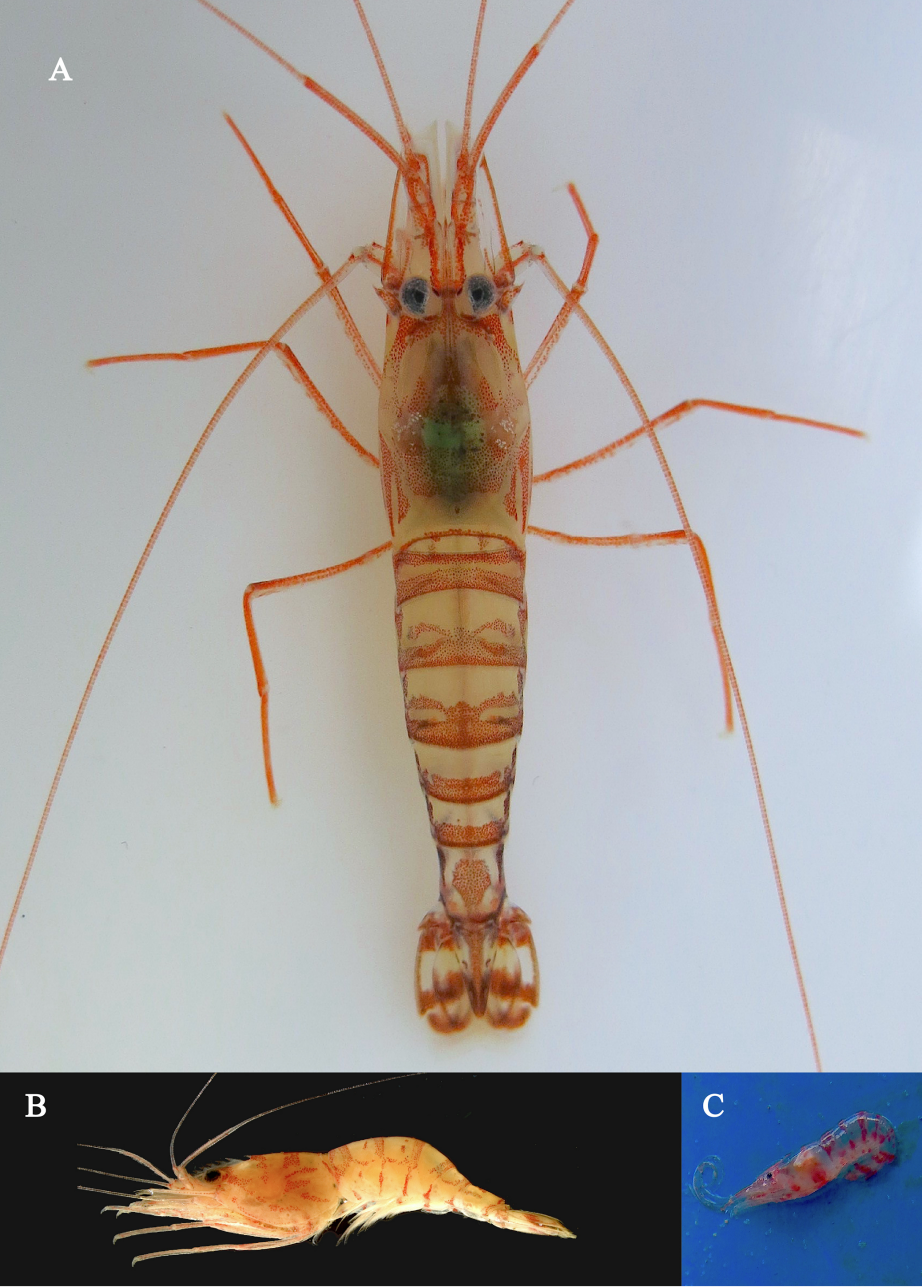
Colors of *L. arvoredensis* sp. nov. (A) holotype (CL+R: 16.2) (MNRJ 27976) after some days in the aquarium; (B) lateral view of paratype 1 fixed just after captured (CL+R 16.60) (LCP/UFSC- 100). Photograph was taken a few days after collection; (C) specimens attached to artificial reef structure (ADCP) immediately before collection. Photographic Credits: (A) Bruno W. Giraldes, (B) Andrea S. Freire and Thais P. Macedo, (C) Alejandro D. Varella.

Specimens were collected in the SW Atlantic Ocean, close to Calhau de São Pedro Islet, Santa Catarina, Brazil (27°25′37.39″S 48°40′11.15″W). A group of specimens of different sizes was captured from an Acoustic Doppler Current Profiler (ADCP) which was deployed at 20 m depth for a few months at the collection site, therefore acting as an artificial reef. Collected specimens were transported alive to the Laboratory of Crustáceos e Plâncton (LCP), Department of Ecologia e Zoologia, Universidade Federal de Santa Catarina (UFSC) for detailed observation of their coloration and color pattern (Fig. 1). Specimens were then preserved in ethanol and studied under a stereomicroscope. The holotype and paratypes were deposited in the National Museum of Rio de Janeiro (MNRJ) and in the Zoological Collection of UFSC (LCP/UFSC). Postorbital carapace length (pocl) and carapace length, including rostrum (cl), were used as measurements of body size and expressed in millimeters.

We were interested in revealing the phylogenetic position of the new species within the genus *Lysmata*. Thus, we conducted molecular phylogenetic analyses using a portion of the 16S mitochondrial DNA fragment. A total of 28 sequences; two sequences from two specimens of *Lysmata arvoredensis* sp. nov. and 27 sequences each from other species belonging to the Neotropical, Cosmopolitan, Cleaner, and Morpho-variable clades of peppermint shrimps were included in the present phylogenetic analyses. Total genomic DNA extraction, PCR amplification with specific 16S rRNA DNA primers (16Sar [5′-CGCCTGTTATCAAAAACAT-3′] and 16Sbr [5′-CCGGTCTGAACTCAGATCACGT-3′] Palumbi, 1996) product cleanup, and sequencing were conducted as described in Baeza et al. (2009). In short, all PCR reactions had a final volume of 20 μL, and contained one μL of the DNA template, one μL of each primer (forward and reverse), and 17 μL of Promega GoTaq Green Master Mix. PCR conditions were initial 95 °C denaturation for 2 min; then 40 cycles of 95 °C denaturation for 30 s, 47 °C annealing for 1 min, and 72 °C extension for 1 min, and a final extension at 72 °C for 5 min. The size and quality of PCR products were visualized on 1.5% agarose gels. PCR products were then purified using Sephadex™ G-50 spin columns. Samples were sequenced using BigDye 3.1 with an automatic sequencer ABI3500 at the Universidade Federal do Paraná (Laboratório de Dinâmica Evolutiva e Sistemas Complexos). Both strands of each sample were sequenced, and then proofread and compiled in Geneious 10.1.3 (Biomatters, Auckland, New Zealand). Alignment of each set of sequences was conducted in MUSCLE (Edgar, 2004) as implemented in MEGA 7.0 (Kumar et al., 2016). The aligned sequences did contain various indels. Thus, we identified positions that were highly divergent and poorly aligned in this 16S gene segment using the software GBlocks v0.91b (Castresana, 2000) and we omitted them from the analyses. After highly divergent positions were pruned, the 16S consisted of 513 bp (88% of the original 582 positions). Next, the aligned gene fragments were analyzed with the software jModelTest 2.1.10 (Guindon & Gascuel, 2003; Darriba et al., 2012) that compares different models of DNA substitution in a hierarchical hypothesis–testing framework to select a base substitution model that best fits each dataset. The optimal model identified by jModelTest (selected with the corrected Akaike Information Criterion, Akaike, 1974) was TPM3uf+G (−lnL = 4280.3454). The calculated parameters were as follows: assumed nucleotide frequencies A = 0.3214, C = 0.1332, G = 0.2067, T = 0.3388; substitution rate matrix with A–C substitution = 0.4427, A–G = 4.7502, A–T = 1.000, C–G = 0.4427, C–T = 4.7502, G–T = 1.0, and rates for variable sites assumed to follow a gamma distribution (G) with shape parameter = 0.2350. Next, we used the webserver W-IQ-TREE (Trifinopoulos et al., 2016, http://iqtree.cibiv.univie.ac.at/) for maximum likelihood (ML) analysis and the software MrBayes (Huelsenbeck & Ronquist, 2001) for Bayesian inference (BI) analysis. As the model selected by jModelTest2 was not available on the webserver W-IQ-TREE (Trifinopoulos et al., 2016, http://iqtree.cibiv.univie.ac.at/), we conducted the ML analysis with the GTR+G evolutionary model that was included within the 95% confidence interval calculated by JModelTest2. All the parameters used for the ML analysis in W-IQ-TREE server were those of the default options. In MrBayes, the analysis was performed for 6,000,000 generations. Every 100th tree was sampled from the MCMC analysis obtaining a total of 60,000 trees and a consensus tree with the 50% majority rule was calculated for the last 59,900 sampled trees. The robustness of the ML tree topologies was assessed by bootstrap reiterations of the observed data 1,000 times. Support for nodes in the BI tree topology was obtained by posterior probability values.

The electronic version of this article in portable document format will represent a published work according to the International Commission on Zoological Nomenclature (ICZN), and hence the new names contained in the electronic version are effectively published under that Code from the electronic edition alone. This published work and the nomenclatural acts it contains have been registered in ZooBank, the online registration system for the ICZN. The ZooBank LSIDs (Life Science Identifiers) can be resolved and the associated information viewed through any standard web browser by appending the LSID to the prefix http://zoobank.org/. The LSID for this publication is: (*L. arvoredensis* sp. nov. urn:lsid:zoobank.org:act:16D1C1E5-DED2-45CF-856F-06A7C167370A; and the publication under urn:lsid:zoobank.org:pub:5ECAB752-E712-42E8-100 8FCA-5C3386D7F7F9). The online version of this work is archived and available from the following digital repositories: PeerJ, PubMed Central, and CLOCKSS.

## Results

### Systematics

**Figure 2:**
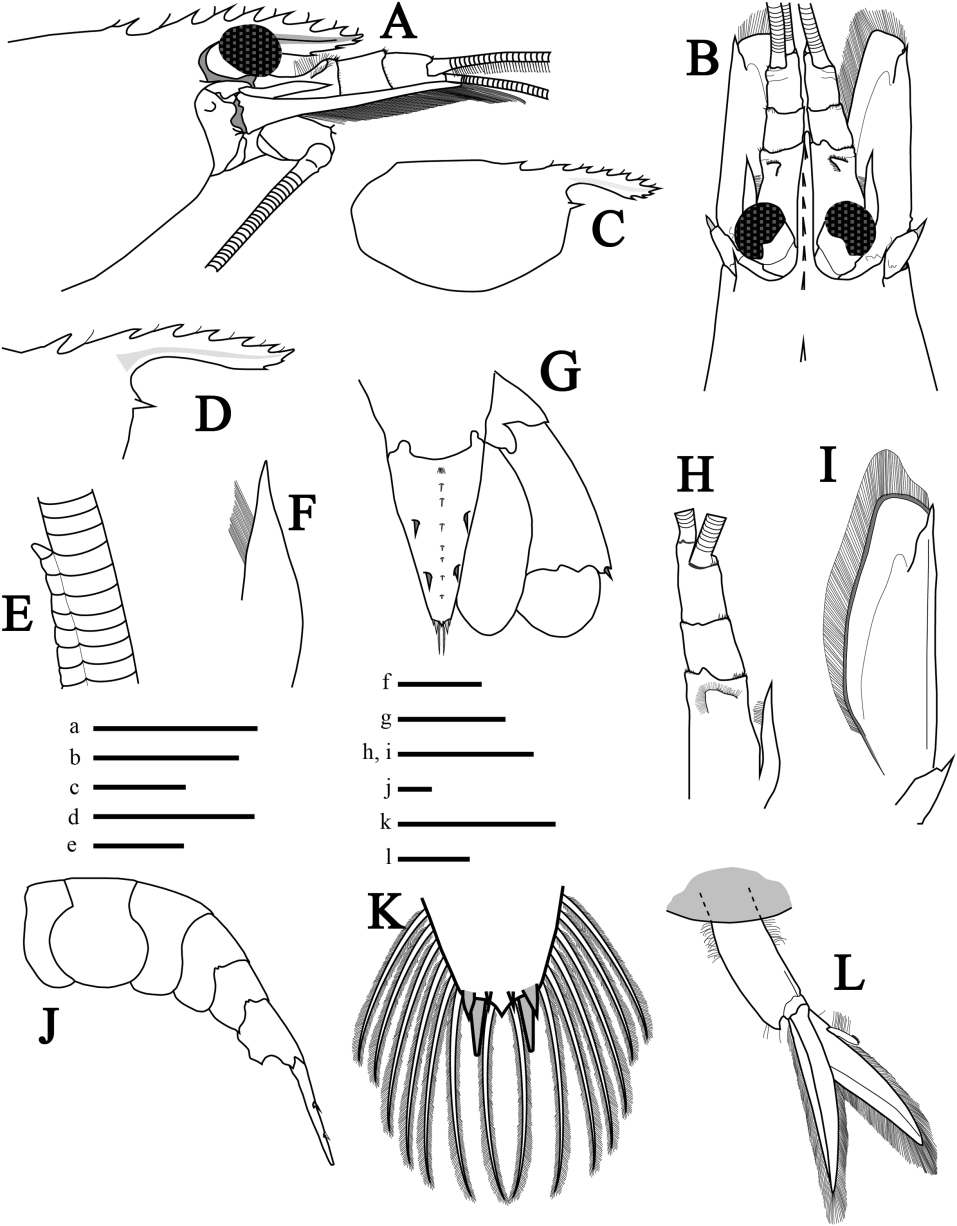
Morphological illustrations of *L. arvoredensis* sp. nov. (A) Frontal region, lateral view; (B) frontal region, dorsal view; (C) total carapace, lateral view; (D) variation of rostrum, lateral view; (E) details of accessory branch in the outer antennular flagellum, lateral view; (F) stylocerite, dorsal view; (G) telson and uropods, dorsal view; (H) antennular peduncle and (I) scaphocerite in same scale, dorsal view; (J) abdominal segments and telson, lateral view; (K) telson, details of posterior margin, dorsal view; (L) second right pleopod, lateral view. Holotype (MNRJ 27976) (A–C, E, G, J–L); Paratype 4 (LCP/UFSC- 104) (D); Paratype 1 (LCP/UFSC- 100) (F, H, I). Scale size, (A–C) five mm; (D, G–J, L) three mm; (E, F, K) one mm. Drawings by Bruno W. Giraldes.

**Figure 3:**
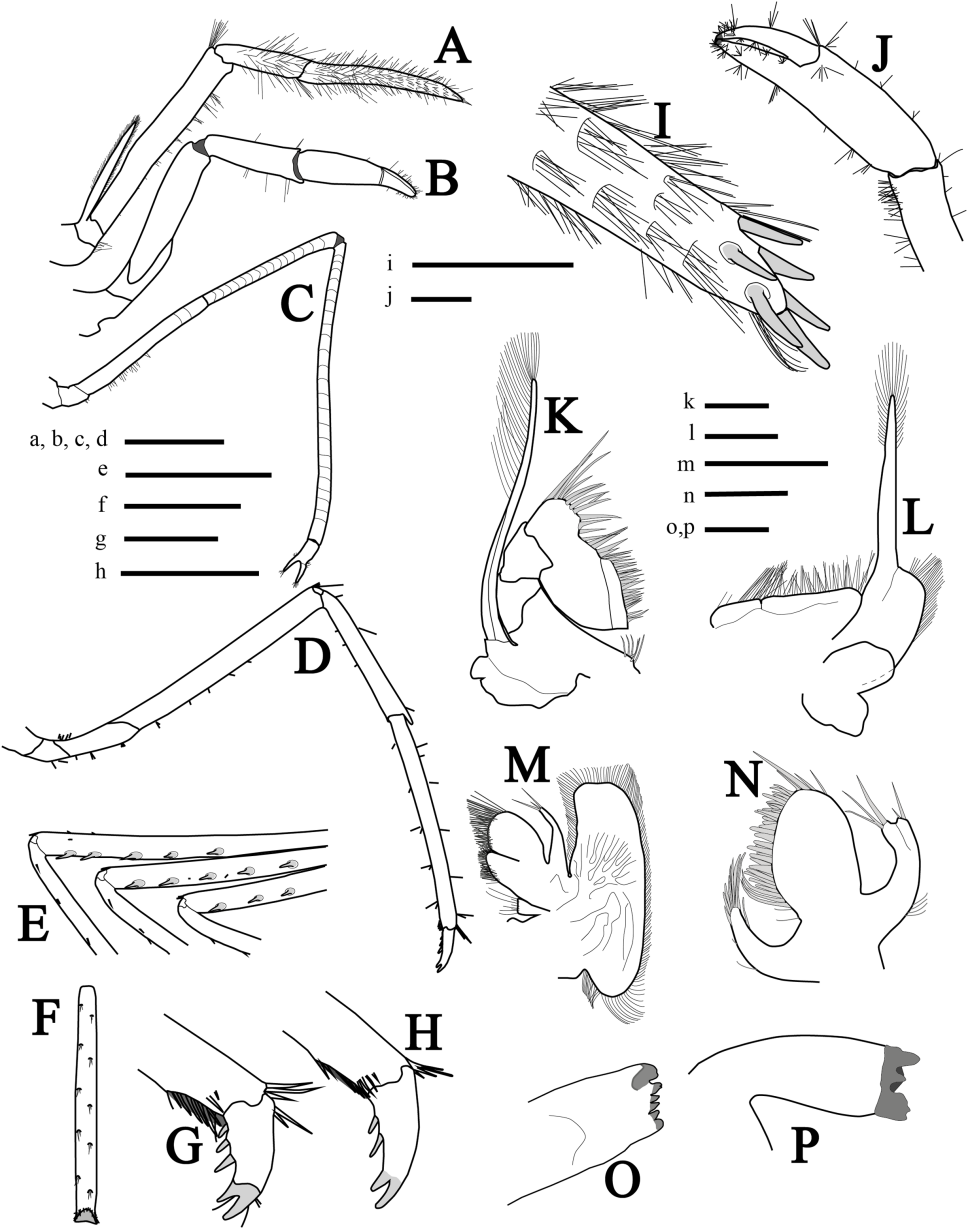
More illustrations of *L. arvoredensis* sp. nov. Lateral/superior view of (A) third maxiliped, (B) first pereiopod, (C) second pereiopod, and (D) third pereiopod; (E) spines in the merus of pereiopods 3, 4, and 5, inferior view; (F) propodus of pereiopod 3, inferior view; (G, H) dactyls of pereiopod 3, lateral view; (I) tip of ultimate segment of the third maxiliped, dorsal view; (J) chela of first pereiopod, inferior view; (K) second maxilliped; (L) first maxilliped; (M) maxilla; (N) maxilulla; (O) right mandible; (P) left mandible. Holotype (MNRJ 27976) (A–G, I–J); Paratype 4 (LCP/UFSC- 104) (H, K–P). Scale size, (A–F) three mm; (G–P) one mm. Drawings by Bruno W. Giraldes.

**Figure 4:**
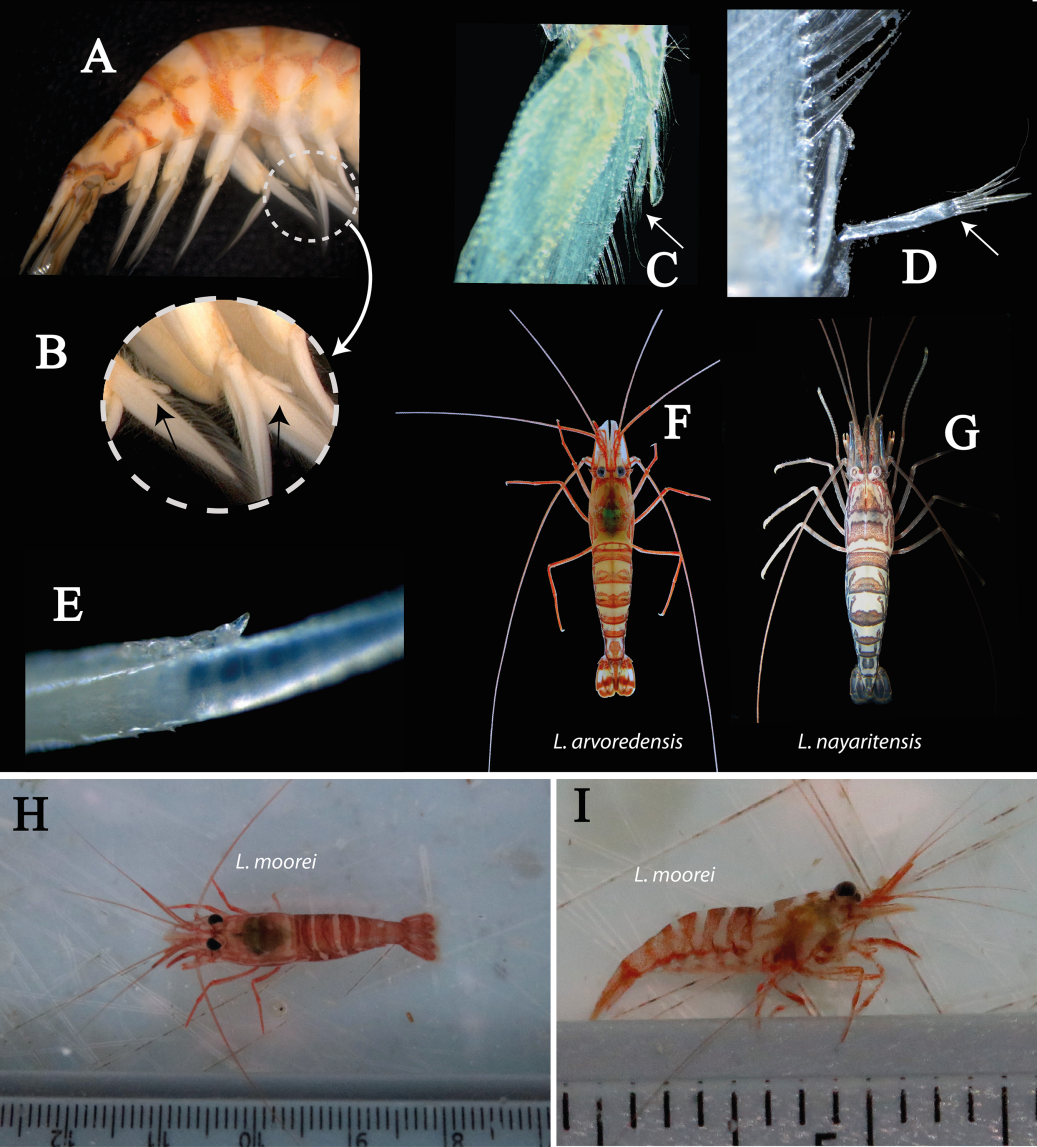
Photography of details of *L. arvoredensis* sp. nov. (A) Abdomen of the hermaphrodite holotype (MNRJ 27976) with details of the (B) endopod of second pleopod (left) and third pleopod (right) lacking appendix masculina; (C, D) second pleopod from small male paratypes 4 and 5 (LCP/UFSC- 104–105) with appendix masculina; (E) the fused segments of the accessory branch with a free unguiform segment in the antennular flagellum. Comparison of color pattern between (F) *L. arvoredensis* sp. nov. and (G) *L. nayaritensis*. Color pattern (H) in dorsal and (I) lateral view of *L. moorei*. Photographic credits: (A, B, F) Bruno W. Giraldes; (C–E) Andrea S. Freire and Thais P. Macedo; (G) J. Antonio Baeza; (H, I) Thais P. Macedo.

Family Lysmatidae Dana, 1852
***Lysmata arvoredensis* sp. nov.**
Figures 1–4

**Material examined.**

**Type material. *Holotype***. adult ovigerous (pocl 11.30, cl 16.20); May 25, 2014; Calhau de São Pedro Island-Santa Catarina, (27°25′37.39″S 48°40′11.15″W), 20 m depth, hidden in a moored current profiler (MNRJ 27976). ***Paratype*s.** One hermaphrodite (pocl 10.50, cl 16.60); May 25, 2014; six males (pocl 3.90–6.30, cl 5.60–9.75); two males (pocl 6.00, 4.65, cl 9.00, 7.00) (muscle extracted for genetic analysis); Calhau de São Pedro Island-Santa Catarina, (27°25′37.39″S 48°40′11.15″W), 20 m depth, hiding in a moored current profiler (LCP/UFSC- 101–107).

**Description of holotype.** Rostrum (Figs. 2A and 2B) short, reaching middle of second segment of antennular peduncle, convex and slightly curved upwards near tip; rostral tip simple, dorsal carina with seven teeth (2+5), posterior-most tooth situated on carapace, with considerable gap between first and second tooth, both anterior to postorbital margin with the second tooth ending just above the postorbital margin; stiff setae among dorsal teeth. Ventral margin with three teeth, proximal ventral tooth not reaching the end of first antennular peduncle, in line with sixth distal dorsal tooth; rostrum length (tip to base of orbit) about 0.46 times that of carapace length. Carapace (Figs. 2A and 2B) smooth, with rounded posteroventral margin; antennal tooth stout, acute, somewhat separated from ventral angle of orbit; pterygostomial angle rounded, pterygostomial tooth absent. Eyes moderately large (Fig. 2B).

Antennular peduncle shorter than scaphocerite blade (Fig. 2B); first segment 0.5 times the length of all antennular peduncle segments, with small setae forming a semicircle located before the distal edge (Fig. 2H). Stylocerite with pointed tip and mesial setae, nearly reaching end of first antennular segment (Figs. 2F and 2H). Second segment of antennular peduncle almost as long as wide; third segment as long as second segment (Fig. 2H). Distomesial angles of antennular segments with spinules (Figs. 2B and 2H). Outer antennular flagellum with aesthetascs extending from first segment to accessory branch; accessory branch nearly completely fused with the outer antennular flagellum bearing only a single free unguiform segment (Figs. 2E and 4E); about 37 joined/fused segments prior to the free unguiform segment.

Antenna with basicerite bearing acute distolateral tooth. Scaphocerite (Figs. 2B and 2I) subrectangular, about 2.7 times as long as wide distally, distolateral tooth stout, acute, falling short of blade distal margin. Scaphocerite slightly longer than antennular peduncle, exceeding distal segment by ~0.25 times its length.

Mouthparts (Figs. 3A, 3I and 3K–3P) as is typical for the genus (Chace, 1972, 1997; Rhyne & Lin, 2006) including unequal but similar mandibles armed with three large incisor teeth on masticatory edge; right with more teeth than left (Figs. 3O and 3P). All maxillipeds with a well-developed exopod (Figs. 3A, 3I, 3K and 3L). Third maxilliped (Figs. 3A and 3I) with dense serrate setae compared to pereiopods, especially on distal segment; overreaching scaphocerite by 1/2 of distal segment; exopod about 0.5 times the length of antepenultimate segment (Fig. 3A); tip of terminal segment armed with stout spines (Fig. 3I).

First pereiopod (P1) (Figs. 3B and 3J) short and robust, overreaching the end of the third maxilliped penultimate segment or reaching the distal margin of scaphocerite. Chela 0.8 times length of carpus, with subcylindrical palm, 1.4 times as long as dactylus (Fig. 3J). Dactylus with corneous tip. Carpus with oblique row of distomesial long setae and ventral surface with sparse setae. Merus 1.5 times as long as carpus and obliquely articulated with ischium (Fig. 3B).

Second pereiopods (P2) (Fig. 3C) long, slender, multi articulated with merus and carpus segmented, right and left subequal in length, ending in simple chela. Chela 5.7 times as long as carpus, with palm 1.4 times as long as dactylus. Carpus 2.0 times as long as merus; and ischium as long as merus. Merus of right and left P2 with 16 and 14 subsegments, respectively; carpus of right and left P2 with 24 and 22 subsegments, respectively; and ischium with three subsegments.

Third to fifth pereiopods similar, decreasing in length from third to fifth. Third pereiopod (Fig. 3D) overreaching distal margin of scaphocerite by proximal third of propodus; merus about six times as long as wide, with five stout ventrolateral spines distally (Fig. 3E), and less than twice as long as carpus; propodus slightly shorter than merus (Fig. 3D), with line of 12 setae on ventral margins; dactylus biunguiculate, about 0.18 times the length of propodus, flexor margin with three spines increasing in size distally (Fig. 3G). Fourth pereiopod similar to third. Fifth pereiopod with merus distinctly shorter than propodus, with three stout ventrolateral spines distally (Fig. 3E); propodus with line of 10 setae on ventral margin (Fig. 3F).

Abdomen (Figs. 2J and 4A) 4.3 times longer than wider (including telson), second pleonite two times wider than sixth; first three pleura with rounded margins laterally, fourth with protruding posterolateral round margin, fifth with sharp posterolateral tooth, sixth with acute posteroventral tooth plus acute posterior tooth on each side of telson (Fig. 2J); sixth segment 1.8 times longer than fifth segment and 1.1 times longer than wider (Fig. 2J). Second and third pleopods with appendix interna (Fig. 2L); endopod in second pleopod lacking appendix masculina (Fig. 4B). Uropod with short protopodite and posterolateral lobe pointed; exopod with diaeresis bearing acute tooth laterally, adjacent to distolateral spine (Fig. 2G); endopod and exopod overreaching the posterior end of telson; exopod slightly longer than endopod, both with plumose setae in lateral and posterior margins.

Telson (Fig. 2G) 1.5 times as long as sixth abdominal somite; tapering posteriorly, about 1.7 times as long as wide at base; dorsal surface with two pairs of stout spines, anterior and posterior pair at ~0.36 and 0.67 of telson length, respectively; longitudinal middle-dorsal line with tufts of setae (Fig. 2G); posterior margin subacute, with pair of longer mesial setae each flanked by shorter lateral setae; lateral and posterior margin of telson with plumose setae (Fig. 2K).

**Variation in paratypes.** Number of dorsal rostral teeth range from 6 to 7, including two postorbital ones. One specimen also exhibited two ventral teeth (Fig. 2D). The number of carpal subsegments in the second pereiopod ranges from 22 to 24. One small specimen with two spines on ventral margin of fifth pereiopod. Propodus in pereiopods 3, 4, and 5 bearing 10–12, 9–12, and 9–12 ventral spines, respectively. Dactyl of pereiopods 3–5 with two spines proximal to biunguiculate tip in two specimens (Fig. 3H). Smaller specimens with appendix interna and appendix masculina in second pleopods (Figs. 4C and 4D). Larger specimens without appendix masculina.

**Color in life.** Color pattern is based on the holotype after remaining a few days in an aquarium (Fig. 1A). Entire body and appendices with semitransparent background and red colored details. Antennas, antennules, and pereiopods red. Antennular scale with red margin. Carapace with irregular oblique bands and patches. No longitudinal red lines in abdomen. Each abdominal segment with narrow transversal band occupying less than half of the surface in dorsal view. Second and third abdominal segments with a dorsal ornament in the shape of a mask formed by chromatophores located immediately anterior to the transversal band. Sixth abdominal segment with a red hexagon in dorsal view. Tail fan ornamented with three transversal bands; uropods with red margins; telson with a red tip (Fig. 1A).

**Type locality.** Calhau de São Pedro Island (27°25′37.39″S 48°40′11.15″W), Santa Catarina, southern Brazil.

**Etymology.** The new species is named after the REBIO Arvoredo, a Marine Protected Area from which the studied specimens were collected.

**Distribution.** Presently known only from the type locality.

**Habitat.** Natural habitat unknown. All specimens were collected from an ADCP, deployed at 20 m for 2 months, acting as an artificial reef substrate. The above suggests that the new species inhabit cavities in hard bottoms.

**Behavior.** That large and small animals were collected together suggests that this species is gregarious.

**Figure 5:**
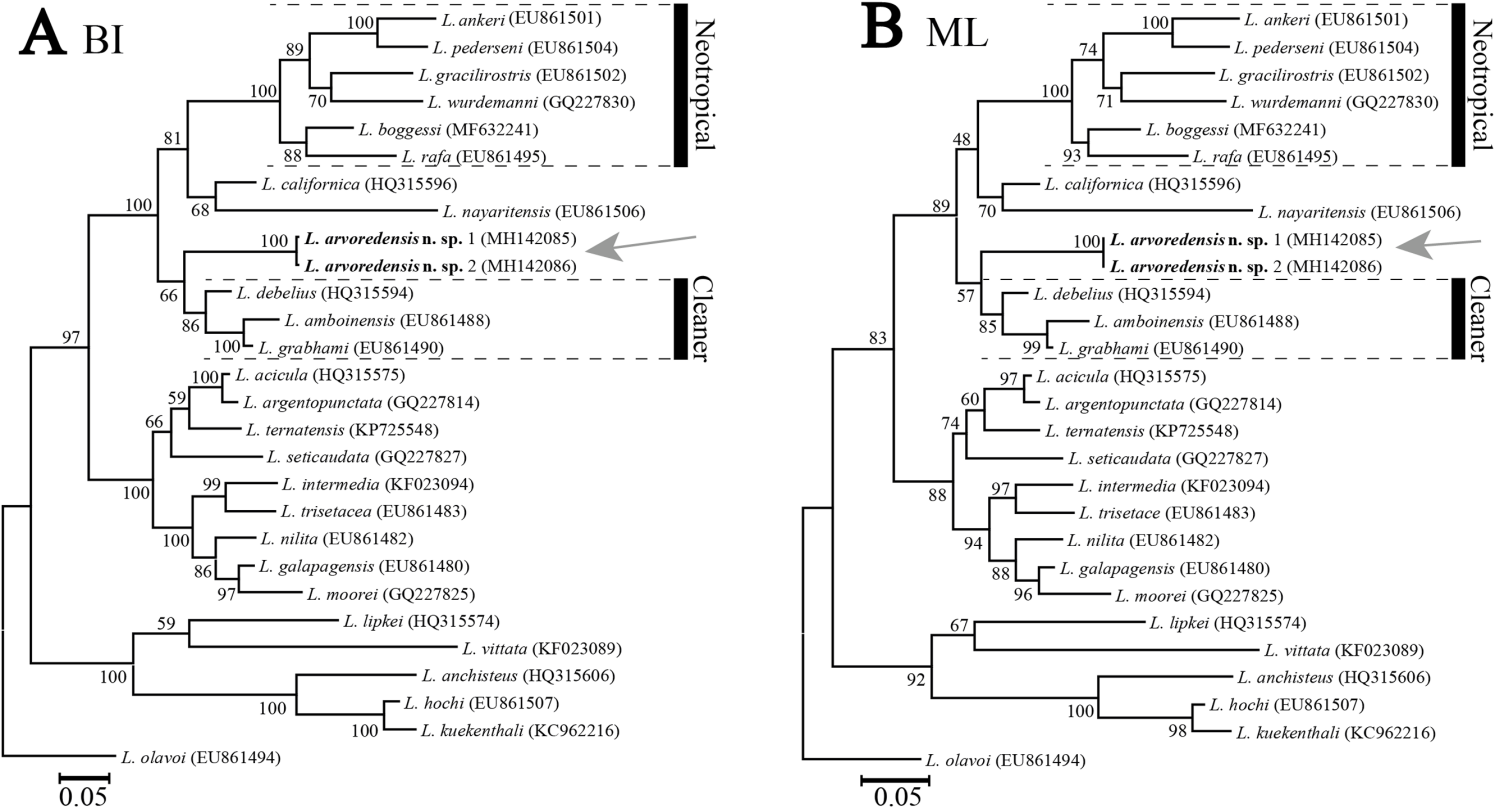
Phylogenetic tree for shrimps from the genus *Lysmata*. Trees were obtained using (A) Bayesian inference (BI) and (B) maximum likelihood (ML) phylogenetic analyses of the partial 16S rRNA mitochondrial DNA gene. Numbers above and/or below the branches represent the bootstrap values obtained from ML and the posterior probability values from the BI analysis (ML/BI). GenBank accession numbers are provided immediately after the species names. Analysis conducted by J. Antonio Baeza and illustrated by Bruno W. Giraldes.

**Phylogenetic results.** The molecular data matrix comprised a total of 513 characters, of which 175 were parsimony informative, for a total of 28 terminals. The analyses conducted with different molecular phylogenetic inference methods (ML and BI) resulted in similar tree topologies (Fig. 5). The new species *L. arvoredensis* sp. nov. (two specimens) clustered together into a monophyletic clade with other species of *Lysmata* belonging to the Neotropical and Cleaner clades (Baeza & Fuentes, 2013).

**Remarks.** This new species increases to 46 the total number of species belonging to the genus *Lysmata* described worldwide (De Grave & Fransen, 2011; Rhyne, Calado & Dos Santos, 2012; Soledade et al., 2013; Gan & Li, 2016; Prakash & Baeza, 2017; De Grave & Anker, 2018) and increases to nine the number of species recorded in Brazil (Chace, 1972; Coelho et al., 2006; Rhyne & Lin, 2006; Soledade et al., 2013; Kassuga, Diele & Hostim-Silva, 2015). *L. arvoredensis* sp. nov. is the fourth species of the genus reported for the temperate waters of Santa Catarina in the south of Brazil (Christoffersen, 1998; Bond-Buckup & Buckup, 1999; Boos et al., 2012; Giraldes & Freire, 2015).

In our phylogenetic analyses, *L. arvoredensis* sp. nov. clustered together and formed a monophyletic clade with other species belonging to the previously recognized Neotropical and Cleaner clades (Baeza et al., 2009). Furthermore, the tree topologies supported a sister relationship between *L. arvoredensis* sp. nov. and congeneric species belonging to the Cleaner clade. However, this relationship was not well supported by both ML and BI analyses. Importantly, *L. arvoredensis* sp. nov. differs considerably both in terms of morphology and coloration from species belonging to the Cleaner clade. All cleaner shrimps exhibit conspicuous color patterns; *L. debelius* Bruce, 1982 has a deep scarlet red coloration in the entire body (Bruce, 1982) while *L. amboinensis* and *L. grabhami* are yellow and possess dorsal longitudinal red and white bands (Hayashi, 1975; Chace, 1997; Baeza, 2010; Kassuga, Diele & Hostim-Silva, 2015). The color pattern of the new species is rather dull in comparison. Furthermore, *L. debelius* has a relatively long rostrum that reaches beyond the intermediate segment of the antennular peduncle, has a dorsal formula of 2+3 teeth, the scaphocerite extends far beyond the antennular peduncle, and the second pereiopod exhibits two subsegments in the merus and 16 in the carpus (Bruce, 1982). In turn, *L. arvoredensis* sp. nov. has a shorter rostrum (just reaching the middle of the intermediate segment of the antennular peduncle), has a dorsal formula of 2+5 teeth, the scaphocerite slightly extends beyond the antennular peduncle, and the second pereiopod has 11–16 subsegments in the merus and 22–24 subsegments in the carpus. *L. debelius* is the only congener recorded with a longitudinal middle-dorsal line of setae on the telson (Bruce, 1982), similar to *L. arvoredensis* sp. nov. (Fig. 2G).

*L. amboinensis* and *L. grabhami* are very similar, having a relatively long rostrum (~0.8 times the carapace length), a pterygostomial spine, a very long antennular peduncle with a first segment ~0.5 as long as the carapace and a second segment twice as long as the third. Also, the stylocerite in *L. amboinensis* and *L. grabhami* is very short, not nearly reaching midlength of the basal segment, and the carpus of the second pereiopod has 19–21 subsegments (Hayashi, 1975; Chace, 1997). In *L. arvoredensis* sp. nov., the rostrum is shorter than in *L. amboinensis* and *L. grabhami* (~0.48 times the carapace length), the pterygostomial spine is absent, the first segment of the antennular peduncle is ~1/3 as long as the carapace, the second segment is short, almost as long as the third segment, the stylocerite reaches the end of the basal segment, and the carpus of the second pereopods have 22–24 subsegments.

*L. nayaritensis* and *L. californica* also exhibit several morphological similarities with the new species, including the accessory branch nearly completely fused with the outer antennular flagellum (Wicksten, 2000). Furthermore, the color pattern of *L. nayaritensis* is very similar to that of *L. arvoredensis* sp. nov. (Fig. 4G). However, *L. nayaritensis* has a relatively long rostrum (0.6 times its carapace length, exceeding the second segment of the antennular peduncle) and a short stylocerite (~0.75 times the length of the first segment of antennular peduncle). In *L. nayaritensis*, the dorsal rostrum formula is 1+5–6, the exopod of the third maxilliped is less than 0.5 times the length of the antepenultimate segment, and the second pereiopod has 15–18 subsegments in the merus. By contrast to *L. nayaritensis*, *L. arvoredensis* sp. nov. has a rostrum (0.48 times the carapace length) that does not reach the end of the second segment of the antennular peduncle. The dorsal rostrum formula is 2+4–5, the stylocerite is relatively long, almost reaching the distal end of the first segment of the antennular peduncle, the exopod of the third maxilliped is 0.5 times the length of the antepenultimate segment, and the second pereiopod has 11–16 subsegments in the merus. With respect to color pattern, *L. arvoredensis* sp. nov. features relatively thin reddish dorsal bars (1/3 of each pleuron) in the second and third abdominal segments, forming a mask (Fig. 4F). In turn, *L. nayaritensis* exhibits thicker dorsal bands in the abdominal segments. Dorsally, in pleonites 2–5, *L. nayaritensis* features two short longitudinal lines of chromatophores located anterior to the band that forms a shape similar to the letter “U” in each segment (Fig. 4G).

*L. californica* presents a pterygostomial tooth, the scaphocerite overreaches the antennular peduncle by nearly the length of the last segment, the spine in the scaphocerite strongly overreaches the blade, and the second pereiopod has more than 25 (25–32 or 27–29) subsegments in the carpus (Chace, 1997; Wicksten, 2000). By contrast to *L. californica*, *L. arvoredensis* sp. nov. does not have a pterygostomial tooth, the scaphocerite slightly overreaches the antennular peduncle, the spine in the scaphocerite does not overreaches the blade, and the second pereiopod has less than 25 (22–24) subsegments in the carpus. The color pattern of *L. californica* is also different from that of *L. arvoredensis.* While *L. arvoredensis* sp. nov. exhibits transversal abdominal bands, *L. californica* bears longitudinal abdominal bands/lines (Wicksten, 2012: 294 plate 2).

*Lysmata arvoredensis* sp. nov. is superficially similar both in terms of color pattern (red abdominal transversal bands) and morphology (unguiform free segment in the accessory branch at the antennular flagellum) to species belonging to the unguiform clade (*sensu* Fiedler et al., 2010) or morpho-variable clade (*sensu* Baeza et al., 2009; Baeza, 2010; Baeza & Fuentes, 2013), including *L. hochi* Baeza & Anker, 2008*, L. kuekenthali* (de Man, 1902), and *L. anchisteus* Chace, 1972. Importantly, our phylogenetic analyses demonstrated that *L. arvoredensis* sp. nov. is genetically dissimilar from the species above. *L. hochi, L. kuekenthali,* and *L. anchisteus* can be easily distinguished from *L. arvoredensis* sp. nov. using a combination of various morphological traits, but most importantly, the dorsal rostral formula (Kubo, 1951; Chace, 1972, 1997; Baeza & Anker, 2008; Soledade et al., 2013). For instance, the dorsal rostrum formula is 2+3 in *L. hochi,* 1+3–4 in *L. kuekenthali,* and 1+4–5 in *L. anchisteus*. By contrast to the species above, the rostral formula is 2+4–5 in *L. arvoredensis* sp. nov.

Lastly, *L. arvoredensis* sp. nov. is similar to *L. uncicornis* Holthuis & Maurin, 1952, a species for which no genetic information exist (Baeza & Anker, 2008; Chace, 1972). However, *L. uncicornis* differs from the new species with respect to the stylocerite that exhibit a series of denticules in the outer margin; the longer first pereiopod that exceeds the scaphocerite by nearly the length of the dactylus; the ventral margin of the propodus in pereiopods 3 and 4 with 6–8 setae, the ventral margin of the propodus in pereiopods 5 with 5 setae, the second pereiopod with a maximum of 14 and 28 subsegments in the merus and carpus, respectively, and the accessory branch of the antennular flagellum that is not fused (not distinguishable) before the unguiform free segment (Holthuis & Maurin, 1952). In *L. arvoredensis* nov. sp., the stylocerite is flat in the outer margin and exhibits mesial setae in the inner margin; the first pereiopod does not exceed the scaphocerite; the ventral margin of the propodus in pereiopods 3, 4, and 5 has 10–12, 9–12, and 9–12 setae, respectively; the second pereiopod has a maximum of 16 and 25 subsegments in the merus and the carpus, respectively, and the accessory branch of the antennular flagellum is fused (distinguishable) before the unguiform free segment.

### Key to species of *Lysmata* from the Southwestern Atlantic Ocean

For *L. vitatta* (senior synonym of *L. rauli*), *L. cf lipkei*, *L. jundalini*, and *L. wurdemanni*, morphological characteristics from specimens collected in the southwestern Atlantic Ocean were used to develop a dichotomous key. Also, the color pattern of *L. moorei* has not been officially described. Here, we used the color pattern observed in specimens collected from tidepools in *Atol da Rocas*, an oceanic island off the Brazilian coast (Figs. 4H and 4I). These specimens were collected and identified by Thais P. Macedo and are deposited at the Zoological Collection of the UFSC (LCP/UFSC- 113). The color pattern exhibited by other species present in Brazil was taken from Gordon (1935), Hayashi (1975), D’Acoz (2000), Rhyne & Lin (2006), Coelho et al. (2006), Baeza (2010), Okuno & Fiedler (2010), Fiedler et al. (2010), Laubenheimer & Rhyne (2010), Rhyne, Calado & Dos Santos (2012), Soledade et al. (2013), Barros-Alves et al. (2015), Giraldes & Freire (2015), Kassuga, Diele & Hostim-Silva (2015), and Terossi et al. (2018). It must be highlighted that the color pattern of most species likely becomes less intense when specimens are subject to high intensity illumination (Wear & Holthuis, 1977; Calvo et al., 2016). As pointed before, we have noticed changes in color intensity depending upon illumination conditions in *L. arvoredensis* sp. nov. (Figs. 1A and 1C).

### Key based on morphology

1—Outer antennular flagellum with accessory branch consisting either of a single unguiform segment or a short segment (two or less articles)2
1—Outer antennular flagellum with accessory branch consisting of more than two free articles8
2—Outer antennular flagellum with accessory branch consisting of an unguiform free segment3
2—Outer antennular flagellum with accessory branch consisting of a short segment (two or less articles)6
3—Stylocerite short, just reaching to midpoint of proximal segment of antennular peduncle, slightly beyond cornea4
3—Stylocerite well developed, overreaching the mid length of proximal segment of antennular peduncle5
4—Second pereiopod with 29–30 subsegments on carpus and 11 on merus. Only one dorsal rostral teeth posterior to the orbit. Antennular peduncle with short second and third segment (Slightly longer than wider)L. wurdemanni
4—Second pereiopod with 15–19 subsegments on carpus and 5–9 on merus. Between 2–4 dorsal rostral teeth posterior to the orbit. Antennular peduncle with longer second and third segments (distinctively much longer than wider)L. vittata
5—Pterygostomial tooth absent. Second pereiopod with 22–24 subsegments on carpus and 11–16 on merus. Short pereiopods, with third pair overreaching the scaphocerite by lengths of dactylus and proximal third of propodus. Shorter scaphocerite, little overreaching distal margin of antennular peduncle*L. arvoredensis* sp. nov.
5—Pterygostomial tooth present. Second pereiopod with 27–32 subsegments on carpus and 23–27 on merus. Long pereiopods, with third pair overreaching the scaphocerite by lengths of dactylus, propodus and distal fourth of carpus. Longer scaphocerite distinctively overreaching distal margin of antennular peduncleL. lipkei
6—Antennular peduncle shorter than scaphocerite; short second segment of antennular peduncle, half-length of first segment. Carpus of second pereiopod with more than 25 subsegments7
6—Antennular peduncle overreaching the scaphocerite; long second segment of antennular peduncle, as long as first segment. Carpus of second pereiopod with 17–23 subsegmentsL. grabhami
7—Longer rostrum 0.6–0.8 times as long as carapace, reaching middle or rarely past the end of third segment of antennular peduncle. Short stylocerite reaching just beyond distal margin of eye, falling well short of end of first segment of antennular peduncle. Carpus of second pereiopod with 33–41 (usually 35–37) subsegmentsL. ankeri
7—Shorter rostrum (about 0.5 times as long as carapace), reaching the middle of second segment of antennular peduncle. Longer stylocerite reaching well beyond level of eye, falling just short of distal margin of first segment of antennular peduncle. Carpus of second pereiopod with 29–31 subsegmentsL. bahia
8—Rostrum and carapace with 6–7 dorsal teeth. Two or three median spines on carapace posterior to rostrum. Carpus of second pereiopod with 28 subsegmentsL. jundalini
8—Rostrum and carapace with 4–5 dorsal teeth. One median spine on carapace posterior to rostrum. Carpus of second pereiopod with 17 subsegmentsL. moorei

### Key based on color in life

1—Color conspicuous consisting of yellow background, two broad dorsolateral bands of a brilliant red separated by a middorsal stripe of white along the entire length of the body. Flagellum of antennae and antennules whiteL. grabhami
1—Without conspicuous pattern. Transparent or semitransparent background body with red lines and bands2
2—Abdomen with the presence of several longitudinal lines/bands in the abdomen4
2—Abdomen with transversal red bands dorsally in the abdomen; with virtual absence of longitudinal lines in the abdomen3
3—Broad transversal bands in the abdomen, covering most of each pleuronL. moorei
3—Narrow transversal bands in the abdomen, occupying less than half of each pleuron*L. arvoredensis* sp. nov.
4—At least one solid transversal band (visible dorsally) in the abdomen among the longitudinal lines/bands5
4—Without defined transversal bands (visible dorsally) in the abdomen; only longitudinal lines/bands8
5—Several large transversal bands dorsally in the abdomen (one per segment); three irregular longitudinal bands running through posterior half of carapace to sixth abdominal somiteL. lipkei
5—Larger transversal band mainly in the third pleuron; absent in most segments6
6—Third pleuron with a broad transverse curved band with a u-shape (dorsal view), forming with the longitudinal lines u-shapes dorsally; several solid and well-defined longitudinal lines in the abdomenL. ankeri
6—Third pleuron with a straight transverse band not directly connected with the longitudinal lines (not forming a u-shape in the dorsal view); with longitudinal lines, but not all solid lines7
7—Longitudinal lines are spotted (general spotted look). Lateral view of abdomen with only longitudinal lines (not diagonal)L. vittata
7—Longitudinal lines are solid. Lateral view of abdomen with diagonal lines connecting with the basal longitudinal lineL. wurdemanni
8—Abdomen with broad irregular sublongitudinal bands and patches; lateral view of abdomens with diagonal bands ventrallyL. bahia
8—Abdomen with longitudinal lines and spotted longitudinal bands; the ventral band in the lateral view is continuous and follow the spotted pattern of other bandsL. jundalini

## Discussion

The dichotomous keys either based on morphological traits or color patterns herein developed aim to support future ecological studies in the south-western Atlantic Ocean. Only a few in situ studies has explored the ecology of the genus *Lysmata* in the region although most species are often observed by scuba divers (Baeza et al., 2016). The genus *Lysmata* exhibits remarkable disparity in terms of ecology, social behavior, and mating systems (Chace, 1997; Rhyne & Lin, 2006; Baeza & Anker, 2008; Baeza et al., 2009; Baeza, 2010; Laubenheimer & Rhyne, 2010; Rhyne, Calado & Dos Santos, 2012; Baeza & Fuentes, 2013; Giraldes, Coelho Filho & Smyth, 2015). Furthermore, species that belong to the Cleaner clade have the ability to remove fish parasites (Rhyne, Lin & Deal, 2004; Karplus, 2014). The above suggests that species in the genus *Lysmata* can serve as bioindicators in reef ecosystems. We argue in favor of additional studies on the ecology and systematics of the species in the south-western Atlantic Ocean to set a baseline with which to monitor environmental health in the region.

## Supplemental Information

10.7717/peerj.5561/supp-1 Supplemental Information 1 GenBank deposition.

## Additional Information and Declarations

### DNA Deposition

The following information was supplied regarding the deposition of DNA sequences: GenBank: BankIt2027186 Seq1 MF380416. BankIt2027186 Seq2 MF380417.

### Data Availability

The following information was supplied regarding data availability: The holotype was deposited in the Natural History Museum of Rio de Janeiro, Brazil (ID: MNRJ 27976).

### New Species Registration

The following information was supplied regarding the registration of a newly described species: Publication LSID: urn:lsid:zoobank.org:pub:5ECAB752-E712-42E8-8FCA-5C3386D7F7F9. *Lysmata arvoredensis*: urn:lsid:zoobank.org:act:16D1C1E5-DED2-45CF-856F-06A7C167370A.
