# Supplementary material for: Lysmata arvoredensis nov. sp. a new species of shrimp from the south coast of Brazil with a key to species of Lysmata (Caridea: Lysmatidae) recorded in the southwestern Atlantic
Source: PeerJ. 2018 Sep 5;6:e5561. doi: 10.7717/peerj.5561 (PMC6129138; doi:10.7717/peerj.5561)
Supplement: Supplemental Information 1 [file peerj-06-5561-s001.pdf]

Assunto:	GenBank MF380416-MF380417
De:	gb-admin@ncbi.nlm.nih.gov (gb-admin@ncbi.nlm.nih.gov)
Para:	manoelacb@yahoo.com.br;
Data:	Segunda-feira, 26 de Junho de 2017 18:42

Dear GenBank Submitter:

Thank you for your direct submission of sequence data to GenBank. We have provided GenBank accession numbers for your nucleotide sequences:

BankIt2027186 Seq1   MF380416
BankIt2027186 Seq2   MF380417

The GenBank accession numbers should appear in any publication that reports or discusses these data, as it gives the community a unique label with which they may retrieve your data from our on-line servers. You may prepare and submit your manuscript before your accessions are released in GenBank.

Submissions are not automatically deposited into GenBank after being accessioned. Each sequence record is individually examined and processed by the GenBank annotation staff to ensure that it is free of errors or problems.

You have not requested a specific release date for your sequence data. Therefore, your record(s) will be released to the public database once they are processed. If this is not what you intended, please contact us as soon as possible with the correct release date.

Since the flatfile record is a display format only and is not an editable format of the data, do not make changes directly to a flatfile. For complete information about different methods to update a sequence record, see: https://www.ncbi.nlm.nih.gov/Genbank/update.html

Any inquiries about your submission should be sent to gb-admin@ncbi.nlm.nih.gov

For more information about the submission process or the available submission tools, please contact GenBank User Support at info@ncbi.nlm.nih.gov.

Please reply using the original subject line.
This will allow for faster processing of your correspondence.

Sincerely,

Rich McVeigh Ph.D.
Contractor

The GenBank Direct Submission Staff
Bethesda, Maryland USA

****
gb-admin@ncbi.nlm.nih.gov (for updates/replies to GenBank entries)
info@ncbi.nlm.nih.gov   (for general questions regarding GenBank)
www.ncbi.nlm.nih.gov/books/NBK51157/ GenBank Submissions Handbook
****

```
LOCUS       Seq1                     626 bp    DNA     linear       23-JUN-2017
DEFINITION  Cytochrome Oxidase Subunit I.
ACCESSION   Seq1
VERSION
SOURCE      mitochondrion Lysmata arvoredensis
  ORGANISM  Lysmata arvoredensis
            Unclassified.
REFERENCE   1  (bases 1 to 626)
  AUTHORS   Macedo,T.P., Brandao,M.C., Giraldes,B.W. and Freire,A.S.
  TITLE     Lysmata arvoredensis nov. sp. (Caridea: Lysmatidae) a new
            peppermint shrimp from southwestern Atlantic Ocean
  JOURNAL   Unpublished
REFERENCE   2  (bases 1 to 626)
  AUTHORS   Macedo,T.P., Brandao,M.C., Giraldes,B.W. and Freire,A.S.
  TITLE     Direct Submission
  JOURNAL   Submitted (23-JUN-2017) Ecology and Zoology, Federal University of
            Santa Catarina, Campus Reitor Joao David Ferreira Lima,
            Florianopolis, Santa Catarina 88040-900, Brazil
COMMENT     Bankit Comment: TAX: Yes, new species/combinations; SEE ATTACHMENT.
            Bankit Comment: TOTAL # OF SEQS:2.

            ##Assembly-Data-START##
            Sequencing Technology :: Sanger dideoxy sequencing
            ##Assembly-Data-END##
FEATURES             Location/Qualifiers
     source          1..626
                     /organism="Lysmata arvoredensis"
                     /organelle="mitochondrion"
                     /mol_type="genomic DNA"
                     /tissue_type="Muscle"
                     /country="Brazil"
BASE COUNT      166 a    133 c    118 g    209 t
ORIGIN
        1 cctgagcagg tatagtagga acatctctca gtcttttaat ccgagctgaa ttaggccaac
       61 caggcagatt aattggaaat gatcaaatct acaacgttat tgttacagct catgcatttg
      121 ttataatttt ttttatagtt ataccaatta taattggggg atttgggaat tgattagttc
      181 cactaatatt aggagcccct gatatagcct ttcctcgtat aaacaacata agattttgac
      241 ttctccctcc ctccttatgt cttctgctgt caagaggtat agtagaaaga ggagtgggga
      301 caggatggac tgtctaccct cctctttcag cagggattgc tcatgcagga gcctctgtag
      361 atatggggat tttctcactc catttagctg gagtctcatc tatcttagga gcagttaact
      421 tcataactac tgttattaac atacgtagct caggtataac aatagaccga ataccttat
      481 ttgtgtgatc tgttttctta acagctattc ttcttctact ttccctacct gtcctagcag
      541 gtgctatcac aatactactt acagatcgaa atttgaacac ttcttttttt gatccagctg
      601 gagggggaga ccccgtcctc taccaa
//
LOCUS       Seq2                     515 bp    DNA     linear       23-JUN-2017
DEFINITION  16S Ribosomal RNA.
ACCESSION   Seq2
VERSION
SOURCE      mitochondrion Lysmata arvoredensis
  ORGANISM  Lysmata arvoredensis
            Unclassified.
REFERENCE   1  (bases 1 to 515)
  AUTHORS   Macedo,T.P., Brandao,M.C., Giraldes,B.W. and Freire,A.S.
  TITLE     Lysmata arvoredensis nov. sp. (Caridea: Lysmatidae) a new
            peppermint shrimp from southwestern Atlantic Ocean
  JOURNAL   Unpublished
REFERENCE   2  (bases 1 to 515)
  AUTHORS   Macedo,T.P., Brandao,M.C., Giraldes,B.W. and Freire,A.S.
  TITLE     Direct Submission
  JOURNAL   Submitted (23-JUN-2017) Ecology and Zoology, Federal University of
            Santa Catarina, Campus Reitor Joao David Ferreira Lima,
            Florianopolis, Santa Catarina 88040-900, Brazil
COMMENT     Bankit Comment: TAX: Yes, new species/combinations; SEE ATTACHMENT.
            Bankit Comment: TOTAL # OF SEQS:2.

            ##Assembly-Data-START##
            Sequencing Technology :: Sanger dideoxy sequencing
            ##Assembly-Data-END##
```

```
FEATURES             Location/Qualifiers
     source          1..515
                     /organism="Lysmata arvoredensis"
                     /organelle="mitochondrion"
                     /mol_type="genomic DNA"
                     /tissue_type="Muscle"
                     /country="Brazil"
BASE COUNT      161 a     70 c    109 g    175 t
ORIGIN
        1 atgtctgtgt gaaaatttaa acatagtctg acctgcccac tgggaaacta aagggccgcg
       61 gtatactgac cgtgcaaagg tagcataatc atttgttctt taattgagga ctggaatgaa
      121 aggtctgaca agaagttagc tgtctctaag ataaatcttt aattttactt ttaagtgaaa
      181 aggcttaaat gcggtagagg ggcgataaga ccctataaaa ctttacaggt ttctagcaat
      241 tatcgaattt ttgtgtaaat ttgtttttag tgttactgtt ttgttggggc gacagaaata
      301 taattaatta actgtttcta gtttaaaaat agttctaatt agttaagttg atcctttaat
      361 agagattaaa agattaagtt actttaggga taacagcgta atttctcttg agagttctaa
      421 tcgacagagt tagttgcgac ctcgatgttg aattaagatg ttatctaggt gcagaagcct
      481 agacagtagg tctgttcgac ctttaaaatc ttacg
//
```
